# Crystal structures of (μ_2_-η^2^,η^2^-4-hydroxybut-2-yn-1-yl 2-bromo-2-methylpropanoate-κ^4^ *C* ^2^,*C* ^3^:*C* ^2^,*C* ^3^)bis[tricarbonylcobalt(II)](*Co—Co*) and [μ_2_-η^2^,η^2^-but-2-yne-1,4-diyl bis(2-bromo-2-methyl­propanoate)-κ^4^ *C* ^2^,*C* ^3^:*C* ^2^,*C* ^3^]bis[tricarbonylcobalt(II)](*Co—Co*)

**DOI:** 10.1107/S1600536814009659

**Published:** 2014-06-23

**Authors:** C. John McAdam, Stephen C. Moratti, Brian H. Robinson, Jim Simpson, Roderick G. Stanley

**Affiliations:** a Department of Chemistry, University of Otago, PO Box 56, Dunedin, New Zealand

**Keywords:** crystal structure, alkynes, alkyne-hexa­carbonyl-dicobalt complex

## Abstract

The title compounds, [Co_2_(C_8_H_11_BrO_3_)(CO)_6_], (1), and [Co_2_(C_12_H_16_Br_2_O_4_)(CO)_6_], (2), result from the replacement of two carbonyl ligands from dicobalt octa­carbonyl by the alkynes 4-hy­droxy­but-2-ynyl 2-bromo-2-methyl­propano­ate and but-2-yne-1,4-diyl bis­(2-bromo-2-methyl­propano­ate), respectively. Both mol­ecules have classic tetra­hedral C_2_Co_2_ cluster cores with the Co^II^ atoms in a highly distorted octa­hedral coordination geometry. The alkyne ligands both adopt a *cis*-bent conformation on coordination. In the crystal structure of (1), classical O—H⋯O and non-classical C—H⋯O contacts form inversion dimers. These combine with weak O⋯O and Br⋯O contacts to stack the mol­ecules into inter­connected columns along the *b*-axis direction. C—H⋯O and C—H⋯Br contacts stabilize the packing for (2), and a weak Br⋯O contact is also observed. Inter­connected columns of mol­ecules again form along the *b-*axis direction.

## Abstract 2

The structures of two alkyne-hexacarbonyl-dicobalt complexes are reported, each with potential ATRP initiator substrates as substituents on the alkynes. The complexes each form tetrahedral C_2_Co_2_ cluster cores with classical sawhorse conformations, while a feature of the crystal packing is the formation of inversion dimers for both molecules.

## Chemical context   

In 1954 alkynes were found to act as ligands and displace two carbonyl groups from dicobalt octa­carbonyl to form alkyne-hexa­carbonyl-dicobalt complexes (Sternberg *et al.*, 1954 ▸). The novelty of these compounds, together with their close isolobal relationship to other members of the ‘tetra­hedrane series’ (Hoffmann, 1982 ▸), spawned enormous inter­est in both the hexa­carbonyls and their substituted derivatives. Applications include use in organic synthesis (Melikyan *et al.*, 2012 ▸), as biological probes (Salmain & Jaouen, 1993 ▸) and in the stab­il­ization of high-performance energetic materials (Windler *et al.*, 2012 ▸). Their diverse redox properties (Robinson & Simpson, 1989 ▸) have also been exploited in the development of mol­ecular wires (McAdam *et al.*, 1996 ▸; Hore *et al.*, 2000 ▸; Xie *et al.*, 2012 ▸) where alkyne-hexa­carbonyl-dicobalt cores are separated by electronically conducting spacers or connecting groups. Our recent inter­est in incorporating redox-active organometallic species into polymer materials (Dana *et al.*, 2007 ▸; McAdam *et al.*, 2008 ▸) prompted us to investigate the synthesis of alkyne-hexa­carbonyl-dicobalt complexes with potential ATRP initiator functionality by the incorporation of one or more known initiator substrates, such as 2-halo-2-methyl propanoyl esters (Wang & Matyjaszewski, 1995 ▸; Laurent & Grayson, 2006 ▸), into the alkyne system. The structures of two such mol­ecules with 2-bromo-2-methyl­propano­ate substituents are reported here.
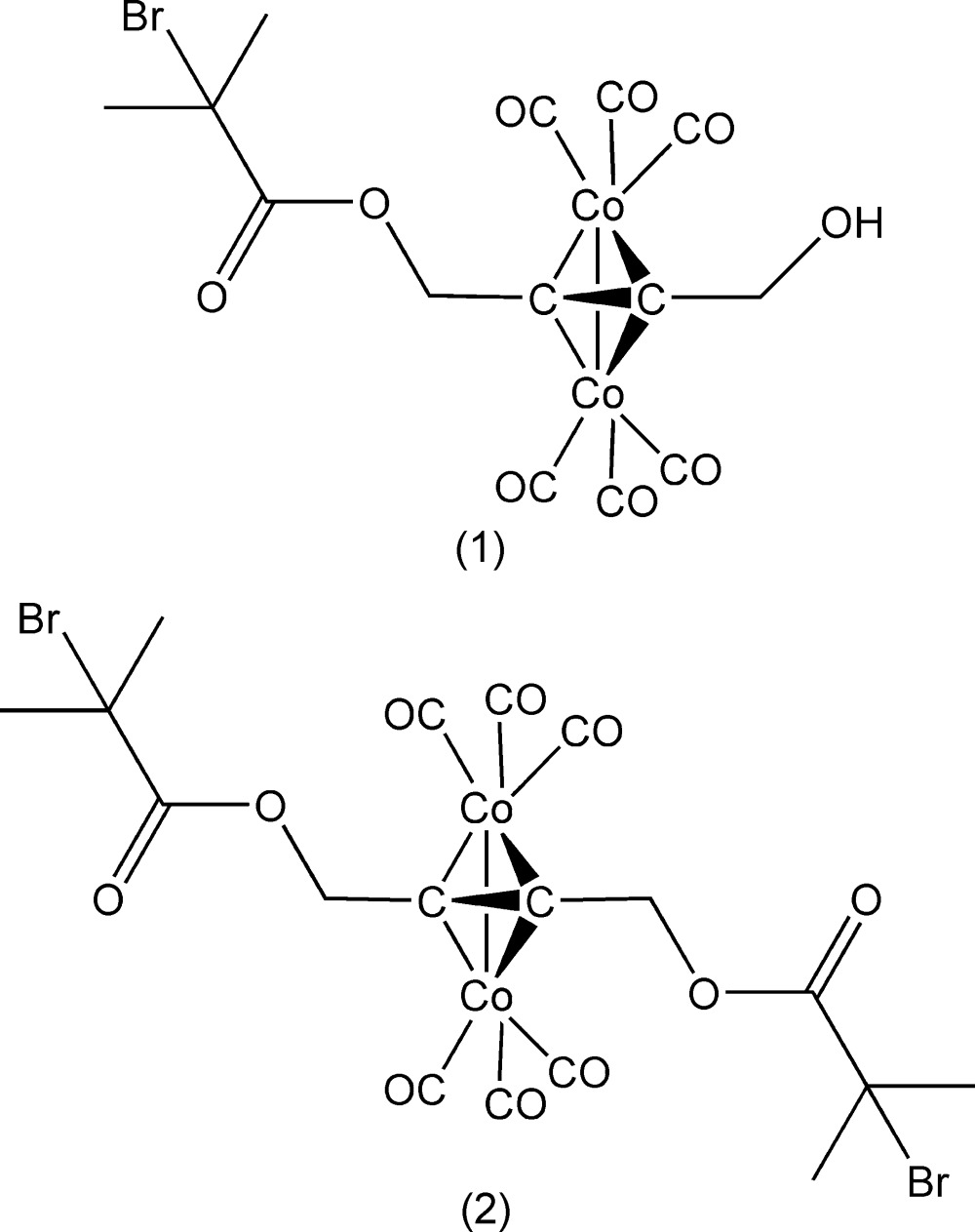


## Structural commentary   

**Figure 1:**
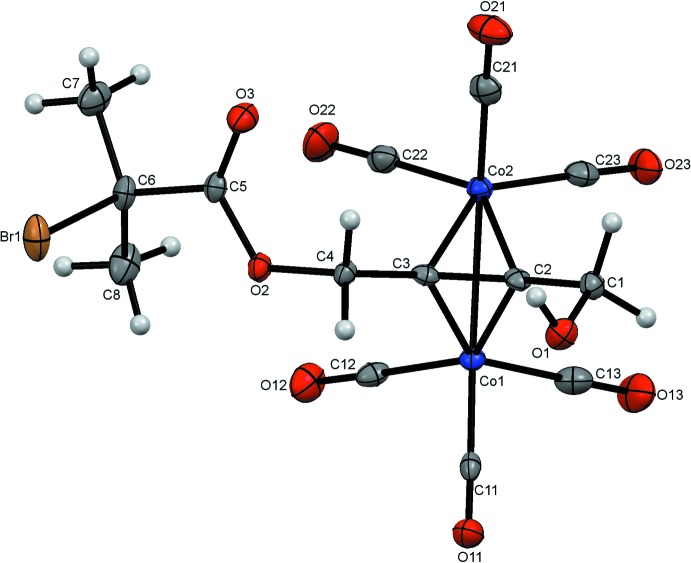
The structure of (1) with ellipsoids drawn at the 50% probability level.

The molecular structures of (1) and (2) are illustrated in Figs. 1 ▸ and 2 ▸. Both compounds are classic alkyne dicobalt cluster systems incorporating the triple bonds of 4-hy­droxy­but-2-ynyl 2-bromo-2-methyl­propano­ate for (1) and but-2-yne-1,4-diyl bis­(2-bromo-2-methyl­propano­ate) for (2) into the tetra­hedral C_2_Co_2_ core of the alkyne dicobalt cluster unit. The coordin­ation geometry around each cobalt atom is distorted octa­hedral. Each cobalt atom carries one pseudo-axial and two pseudo-equatorial carbonyl substituents. The C2 and C3 atoms of the alkyne ligand for (1) and the corresponding C1 and C2 atoms for (2) are also pseudo-equatorial, with the bonds to the second Co atoms completing the highly distorted coordination spheres in pseudo-axial sites.

This combination of coordination spheres results in classical ‘sawhorse’ structures (Arewgoda *et al.*, 1983 ▸) for each mol­ecule. The CH_2_OH and 2-bromo-2-ethyl­propano­ate substit­uents for (1) and the two 2-bromo-2-ethyl­propano­ate groups for (2), adopt a *cis*-bent configuration similar to the excited state of an alkyne system (Dickson & Fraser, 1974 ▸). Furthermore, the C11—Co1—Co2—C21 and C1—C2—C3—C4 planes for (1) and C15—Co1—Co2—C18 and C3—C2—C1—C8 planes for (2) are close to orthogonal with inter­planar angles of 89.65 (7) and 85.91 (7)°, respectively. The Co1—Co2 bond lengths are 2.4723 (7) Å for (1) and 2.4759 (10) Å for (2) with corresponding C2—C3 and C1—C2 distances of 1.344 (5) and 1.343 (3) Å (Tables 1 ▸ and 2 ▸). These are not unusual in comparison to those found for the 480 C_2_Co_2_ alkyne dicobalt clusters with 6 CO ligands found in the Cambridge Structural Database (Allen, 2002 ▸). For these, the mean Co—Co and C—C distances are found to be 2.47 (1) and 1.337 (15) Å, respectively. The eight Co—C_alkyne_ distances average 1.958 (7) Å, again comparable to the mean value of 1.965 (5) Å found previously.

The C=O groups of the 2-bromo-2-methyl­propano­ate units point away from the cluster cores in both mol­ecules. The two carbonyl groups in (2) each lie on the same side of the mol­ecule, with the 2-bromo-2-methyl­propano­ate units arranged symmetrically with respect to the central C_2_Co_2_ unit. Bond lengths (Allen *et al.*, 1987 ▸) and angles in the –OC(O)–C(CH_3_)_2_Br chains are not unusual and are similar in both mol­ecules.

## Supra­molecular features   

**Figure 3:**
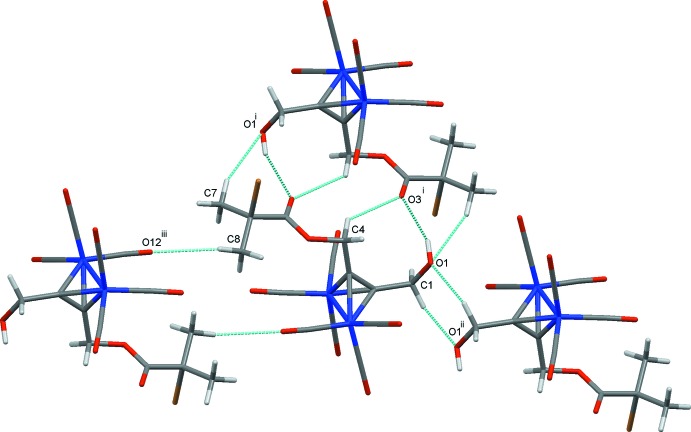
Inversion dimers in the crystal structure of (1). Hydrogen bonds are drawn as dashed lines and symmetry operations are those detailed in Table 2 ▸.

**Figure 4:**
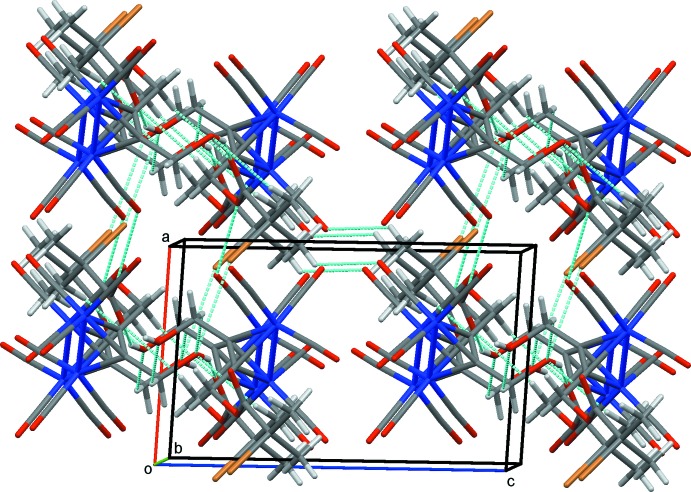
Overall packing for (1) viewed along the *b* axis. Hydrogen bonds and other inter­atomic contacts are drawn as dashed lines.

In the crystal structure of (1), classical O1—H1⋯O3 hydrogen bonds (Table 3 ▸) are augmented by two C—H⋯O contacts that link adjacent mol­ecules into inversion dimers generating 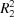
(10), 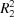
(18)and 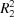
(20) rings (Bernstein *et al.*, 1995 ▸). Two additional inversion dimers also result from weaker C1—H1*A*⋯O1 and C8—H8*A*⋯O12 hydrogen bonds (Fig. 3 ▸). These contacts, together with weak O2⋯O21, [2.965 (4) Å; symmetry operation 1 + *x*, *y*, *z*) and Br1⋯O1 [3.307 (3) Å; symmetry operation −*x*, 1 − *y*, 2 − *z*] contacts stack the mol­ecules into inter­connected columns along the *b*-axis direction (Fig. 4 ▸).

**Figure 5:**
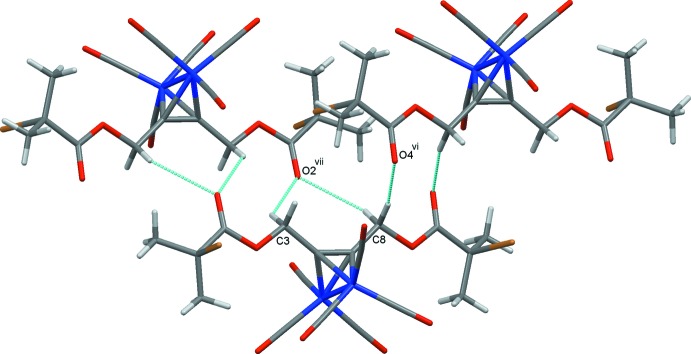
C—H⋯O hydrogen bonds in the crystal structure of (2). Hydrogen bonds are drawn as dashed lines and symmetry operations are those detailed in Table 4 ▸.

**Figure 6:**
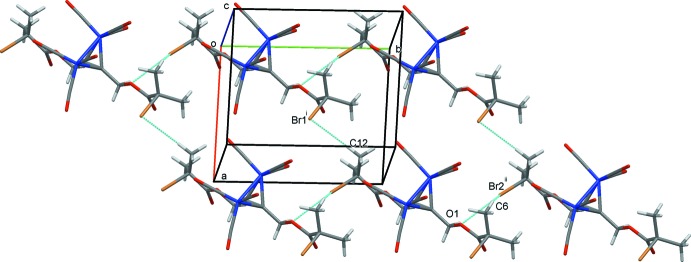
Chains of mol­ecules of (2) formed by C—H⋯Br hydrogen bonds drawn as dashed lines. Symmetry operations are those detailed in Table 4 ▸.

**Figure 7:**
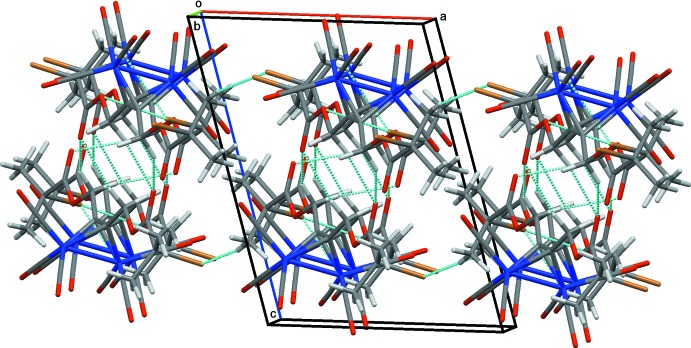
Overall packing for (2) viewed along the *b* axis. Hydrogen bonds and other inter­atomic contacts are drawn as dashed lines.

Hydrogen bonding also figures prominently in the structure of (2), although in this mol­ecule no classical hydrogen bonds are possible. Bifurcated C3—H3*B*⋯O2 and C8–H8*A*⋯O2 contacts (Table 4 ▸) produce 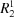
(7) rings while inversion-related C8—H8*B*⋯O4 hydrogen bonds form 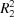
(10) rings (Fig. 5 ▸). The other significant contacts involve C—H⋯Br hydrogen bonds. C12—H12*C*⋯Br1 contacts link mol­ecules into 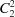
(14) chains approximately parallel to [110] while C6—H6*A*⋯Br2 inter­actions, bolstered by short O1⋯Br2 contacts [3.296 (2) Å, symmetry operation *x*, −1 + *y*, *z*], form 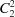
(12) chains parallel to [010] (Fig. 6 ▸). The net result of these contacts is a series of inter­connected columns of mol­ecules stacked along the *b*-axis direction (Fig. 7 ▸).

## Database survey   

The first structure, of dicobalt hexa­carbonyl di­phenyl­acetyl­ene, was reported using film data (Sly, 1959 ▸). The current database (Version 5.35, November 2013 with 1 update) details 480 hexa­carbonyl structures. However, this number rises to 730 if the search is extended to cover dicobalt alkyne compounds in which one or more carbonyl group has been substituted, mainly by phosphine ligands. Inter­estingly there are no current examples of similar 4-hydroxybut-2-ynyl carboxylate derivatives and only one but-2-yne-1,4-diyl di­ace­tate complex [(4-di­acet­oxy­but-2-yne)-hexa­carbonyl-dicobalt; Soleilhavoup *et al.*, 2002 ▸] among this plethora of structures, underlining the novelty of the compounds reported here.

## Synthesis and crystallization   

In typical preparations, 1:1 molar qu­anti­ties of 4-hy­droxy­but-2-ynyl 2-bromo-2-methyl­propano­ate for (1) or a 2:1 molar ratio of but-2-yne-1,4-diyl bis­(2-bromo-2-methyl­propano­ate) for (2) with Co_2_(CO)_8_ were allowed to react at room temperature for 1 h in CH_2_Cl_2_ under nitro­gen. The reaction mixtures were filtered through silica gel to remove any insol­uble impurities and the filtrates taken to dryness *in vacuo*. The complexes were then purified by recrystallization from hexane at 273 K. Yields were in the range 70–80%. Complexation was confirmed by the absence of a band at 1860 cm^−1^ in the infrared spectrum, attributable to the μ_2_ (bridging) carbonyl groups of the dicobalt octa­carbonyl starting material. In addition, a hypsochromic shift of approximately 30 cm^−1^ of the remaining carbonyl stretching frequencies is seen, due to the decrease in electron density at the metal atoms upon coordination of these alkynes. Characteristic IR spectra were recorded for both products as follows: IR (ν, cm^−1^): (1): 3300 (broad, OH), ν(C≡O) 2099, 2062, 2032, ν(C=O) 1735; (2): ν(C≡O) 2096, 2058, 2031, ν(C=O) 1734.

## Refinement   

All H atoms bound to carbon were refined using a riding model with *d*(C—H) = 0.99 Å, *U*_iso_ = 1.2*U*_eq_ (C) for CH_2_, 0.98 Å, *U*_iso_ = 1.5*U*_eq_ (C) for CH_3_ atoms. In the final refinement, two reflections from the data for (2) with *F*_o_ << *F*_c_ were omitted from the refinement.

## Supplementary Material

Crystal structure: contains datablock(s) global, 1, 2. DOI: 10.1107/S1600536814009659/hb0001sup1.cif

Structure factors: contains datablock(s) 1. DOI: 10.1107/S1600536814009659/hb00011sup2.hkl

Structure factors: contains datablock(s) 2. DOI: 10.1107/S1600536814009659/hb00012sup3.hkl

CCDC references: 1004257, 1004258

Additional supporting information:  crystallographic information; 3D view; checkCIF report

## Figures and Tables

**Figure 2:**
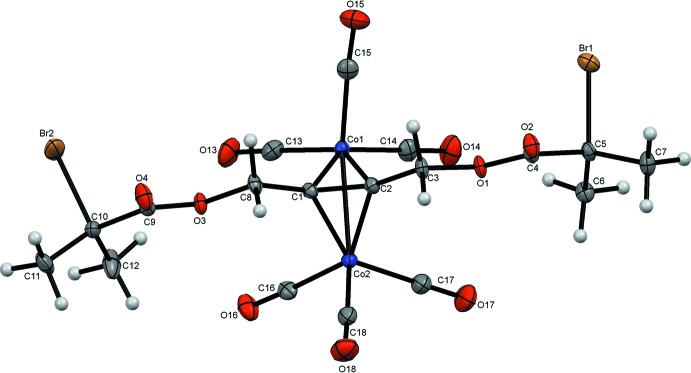
The structure of (2) with ellipsoids drawn at the 50% probability level.

**Table 1:** Selected bond lengths (Å) for (1)

C2—C3	1.344 (5)	C2—Co2	1.972 (3)
Co1—Co2	2.4723 (7)	C3—Co1	1.956 (4)
C2—Co1	1.967 (3)	C3—Co2	1.960 (3)

**Table 2:** Selected bond lengths (Å) for (2)

C1—C2	1.343 (3)	C1—Co2	1.949 (2)
Co1—Co2	2.4759 (10)	C2—Co1	1.9508 (19)
C1—Co1	1.960 (2)	C2—Co2	1.948 (2)

**Table 3:** Hydrogen-bond geometry (Å, °) for (1)

*D*—H⋯*A*	*D*—H	H⋯*A*	*D*⋯*A*	*D*—H⋯*A*
O1—H1⋯O3^i^	0.84	2.16	2.946 (4)	156
C4—H4*B*⋯O3^i^	0.99	2.60	3.360 (4)	134
C7—H7*A*⋯O1^i^	0.98	2.71	3.637 (5)	157
C1—H1*A*⋯O1^ii^	0.99	2.55	3.307 (5)	133
C8—H8*A*⋯O12^iii^	0.98	2.71	3.485 (5)	136

Symmetry codes: (i) 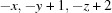; (ii) 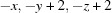; (iii) 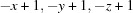.

**Table 4:** Hydrogen-bond geometry (Å, °) for (2)

*D*—H⋯*A*	*D*—H	H⋯*A*	*D*⋯*A*	*D*—H⋯*A*
C12—H12*C*⋯Br1^i^	0.98	2.99	3.961 (3)	170
C6—H6*A*⋯Br2^ii^	0.98	3.01	3.788 (2)	137
C8—H8*B*⋯O4^iii^	0.99	2.45	3.411 (3)	165
C3—H3*B*⋯O2^iv^	0.99	2.58	3.341 (3)	133
C8—H8*A*⋯O2^iv^	0.99	2.64	3.454 (3)	139

Symmetry codes: (i) 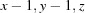; (ii) 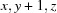; (iii) 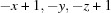; (iv) 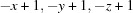.

**Table 5:** Experimental details

	(1)	(2)
Crystal data		
Chemical formula	[Co_2_(C_8_H_11_BrO_3_)(CO)_6_]	[Co_2_(C_12_H_16_Br_2_O_4_)(CO)_6_]
*M*_r_	521.00	669.99
Crystal system, space group	Triclinic, *P* 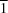	Triclinic, *P* 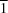
Temperature (K)	91	91
*a*, *b*, *c* (Å)	7.3887 (8), 11.1147 (12), 11.7274 (13)	9.392 (5), 10.710 (5), 13.269 (5)
α, β, γ (°)	78.583 (6), 85.239 (6), 76.342 (6)	71.314 (5), 71.973 (5), 84.630 (5)
*V* (Å^3^)	916.67 (18)	1202.3 (10)
*Z*	2	2
Radiation type	Mo *K*α	Mo *K*α
μ (mm^−1^)	4.03	4.75
Crystal size (mm)	0.39 × 0.16 × 0.04	0.25 × 0.11 × 0.06

Data collection		
Diffractometer	Bruker APEXII CCD area detector	Bruker APEXII CCD area detector
Absorption correction	Multi-scan (*SADABS*; Bruker, 2011 ▸)	Multi-scan (*SADABS*; Bruker, 2011 ▸)
*T*_min_, *T*_max_	0.302, 0.855	0.611, 1.000
No. of measured, independent and observed [*I* > 2σ(*I*)] reflections	11686, 3713, 2966	21546, 8127, 6040
*R*_int_	0.055	0.037
(sin θ/λ)_max_ (Å^−1^)	0.628	0.751

Refinement		
*R*[*F* ^2^ > 2σ(*F* ^2^)], *wR*(*F* ^2^), *S*	0.038, 0.097, 1.03	0.031, 0.069, 0.95
No. of reflections	3713	8127
No. of parameters	238	293
H-atom treatment	H-atom parameters constrained	H-atom parameters constrained
Δρ_max_, Δρ_min_ (e Å^−3^)	0.83, −0.73	1.43, −1.06

Computer programs: *APEX2* and *SAINT* (Bruker, 2011 ▸), *SHELXS97* and *SHELXL2013* (Sheldrick, 2008 ▸), *TITAN2000* (Hunter & Simpson, 1999 ▸), *Mercury* (Macrae *et al.*, 2008 ▸), *enCIFer* (Allen *et al.*, 2004 ▸), *PLATON* (Spek, 2009 ▸) and *publCIF* (Westrip, 2010 ▸).

## supplementary crystallographic information

### (1) [µ2-η2,η2-4-Hydroxybut-2-yn-1-yl 2-bromo-2-methylpropanoate-κ4C2,C3:C2,C3]bis[tricarbonylcobalt(II)](Co—Co) Crystal data

**Table:**

[Co_2_(C_8_H_11_BrO_3_)(CO)_6_]	*Z* = 2
*M**_r_* = 521.00	*F*(000) = 512
Triclinic, *P*1	*D*_x_ = 1.888 Mg m^−^^3^
*a* = 7.3887 (8) Å	Mo *K*α radiation, λ = 0.71073 Å
*b* = 11.1147 (12) Å	Cell parameters from 3089 reflections
*c* = 11.7274 (13) Å	θ = 4.7–51.2°
α = 78.583 (6)°	µ = 4.03 mm^−^^1^
β = 85.239 (6)°	*T* = 91 K
γ = 76.342 (6)°	Irregular fragment, orange-red
*V* = 916.67 (18) Å^3^	0.39 × 0.16 × 0.04 mm

### (1) [µ2-η2,η2-4-Hydroxybut-2-yn-1-yl 2-bromo-2-methylpropanoate-κ4C2,C3:C2,C3]bis[tricarbonylcobalt(II)](Co—Co) Data collection

**Table:**

Bruker APEXII CCD area-detector diffractometer	3713 independent reflections
Radiation source: fine-focus sealed tube	2966 reflections with *I* > 2σ(*I*)
Graphite monochromator	*R*_int_ = 0.055
ω scans	θ_max_ = 26.5°, θ_min_ = 3.3°
Absorption correction: multi-scan (SADABS; Bruker, 2011)	*h* = −8→9
*T*_min_ = 0.302, *T*_max_ = 0.855	*k* = −13→13
11686 measured reflections	*l* = −14→14

### (1) [µ2-η2,η2-4-Hydroxybut-2-yn-1-yl 2-bromo-2-methylpropanoate-κ4C2,C3:C2,C3]bis[tricarbonylcobalt(II)](Co—Co) Refinement

**Table:**

Refinement on *F*^2^	0 restraints
Least-squares matrix: full	Hydrogen site location: inferred from neighbouring sites
*R*[*F*^2^ > 2σ(*F*^2^)] = 0.038	H-atom parameters constrained
*wR*(*F*^2^) = 0.097	*w* = 1/[σ^2^(*F*_o_^2^) + (0.0486*P*)^2^] where *P* = (*F*_o_^2^ + 2*F*_c_^2^)/3
*S* = 1.03	(Δ/σ)_max_ = 0.001
3713 reflections	Δρ_max_ = 0.83 e Å^−^^3^
238 parameters	Δρ_min_ = −0.73 e Å^−^^3^

### (1) [µ2-η2,η2-4-Hydroxybut-2-yn-1-yl 2-bromo-2-methylpropanoate-κ4C2,C3:C2,C3]bis[tricarbonylcobalt(II)](Co—Co) Special details

**Table:**

Geometry. All e.s.d.'s (except the e.s.d. in the dihedral angle between two l.s. planes) are estimated using the full covariance matrix. The cell e.s.d.'s are taken into account individually in the estimation of e.s.d.'s in distances, angles and torsion angles; correlations between e.s.d.'s in cell parameters are only used when they are defined by crystal symmetry. An approximate (isotropic) treatment of cell e.s.d.'s is used for estimating e.s.d.'s involving l.s. planes.

### (1) [µ2-η2,η2-4-Hydroxybut-2-yn-1-yl 2-bromo-2-methylpropanoate-κ4C2,C3:C2,C3]bis[tricarbonylcobalt(II)](Co—Co) Fractional atomic coordinates and isotropic or equivalent isotropic displacement parameters (Å^2^)

**Table:**

	*x*	*y*	*z*	*U*_iso_*/*U*_eq_	
O1	−0.0205 (4)	0.8525 (2)	1.0602 (2)	0.0210 (6)	
H1	−0.0387	0.7844	1.1000	0.032*	
C1	−0.1326 (5)	0.8873 (4)	0.9601 (3)	0.0159 (8)	
H1A	−0.1616	0.9800	0.9357	0.019*	
H1B	−0.2517	0.8605	0.9803	0.019*	
C2	−0.0346 (4)	0.8277 (3)	0.8618 (3)	0.0137 (7)	
C3	0.0896 (4)	0.7232 (3)	0.8429 (3)	0.0135 (7)	
C4	0.1996 (5)	0.6065 (3)	0.9107 (3)	0.0131 (7)	
H4A	0.2813	0.6282	0.9626	0.016*	
H4B	0.1144	0.5594	0.9600	0.016*	
O2	0.3132 (3)	0.5272 (2)	0.8345 (2)	0.0160 (5)	
C5	0.2596 (5)	0.4240 (3)	0.8234 (3)	0.0147 (8)	
O3	0.1198 (4)	0.3939 (2)	0.8680 (2)	0.0220 (6)	
C6	0.3981 (5)	0.3471 (3)	0.7453 (3)	0.0185 (8)	
C7	0.3045 (6)	0.2640 (4)	0.6933 (3)	0.0254 (9)	
H7A	0.2510	0.2089	0.7560	0.038*	
H7B	0.3967	0.2127	0.6472	0.038*	
H7C	0.2052	0.3169	0.6431	0.038*	
C8	0.5019 (6)	0.4259 (4)	0.6532 (3)	0.0274 (9)	
H8A	0.5921	0.3703	0.6097	0.041*	
H8B	0.5676	0.4733	0.6910	0.041*	
H8C	0.4126	0.4847	0.5995	0.041*	
Br1	0.58507 (6)	0.23943 (4)	0.85597 (4)	0.02956 (14)	
Co1	0.14649 (6)	0.86999 (5)	0.73644 (4)	0.01350 (13)	
C11	0.3041 (5)	0.9142 (3)	0.8218 (3)	0.0153 (8)	
O11	0.4032 (3)	0.9404 (3)	0.8748 (2)	0.0216 (6)	
C12	0.3119 (5)	0.7980 (4)	0.6321 (3)	0.0188 (8)	
O12	0.4169 (4)	0.7477 (3)	0.5703 (2)	0.0299 (7)	
C13	0.0360 (5)	1.0278 (4)	0.6608 (3)	0.0235 (9)	
O13	−0.0335 (4)	1.1244 (3)	0.6144 (3)	0.0358 (8)	
Co2	−0.11056 (6)	0.76340 (5)	0.73243 (4)	0.01475 (14)	
C21	−0.2640 (5)	0.6741 (4)	0.8176 (3)	0.0216 (9)	
O21	−0.3575 (4)	0.6190 (3)	0.8772 (3)	0.0327 (7)	
C22	−0.0153 (5)	0.6651 (4)	0.6246 (3)	0.0216 (9)	
O22	0.0493 (4)	0.6009 (3)	0.5607 (3)	0.0342 (7)	
C23	−0.2750 (5)	0.8974 (4)	0.6540 (3)	0.0216 (8)	
O23	−0.3745 (4)	0.9823 (3)	0.6055 (2)	0.0293 (7)	

### (1) [µ2-η2,η2-4-Hydroxybut-2-yn-1-yl 2-bromo-2-methylpropanoate-κ4C2,C3:C2,C3]bis[tricarbonylcobalt(II)](Co—Co) Atomic displacement parameters (Å^2^)

**Table:**

	*U*^11^	*U*^22^	*U*^33^	*U*^12^	*U*^13^	*U*^23^
O1	0.0248 (14)	0.0207 (15)	0.0180 (14)	−0.0051 (12)	−0.0019 (11)	−0.0039 (11)
C1	0.0151 (17)	0.0157 (19)	0.0168 (19)	−0.0025 (15)	0.0022 (14)	−0.0049 (15)
C2	0.0112 (17)	0.0148 (19)	0.0147 (18)	−0.0052 (14)	0.0024 (13)	−0.0003 (15)
C3	0.0114 (16)	0.0169 (19)	0.0132 (18)	−0.0063 (14)	0.0017 (13)	−0.0020 (15)
C4	0.0151 (17)	0.0108 (18)	0.0135 (18)	−0.0024 (14)	0.0019 (14)	−0.0040 (14)
O2	0.0163 (12)	0.0133 (13)	0.0174 (13)	−0.0020 (10)	0.0043 (10)	−0.0041 (11)
C5	0.0173 (18)	0.0119 (18)	0.0126 (18)	−0.0001 (14)	−0.0014 (14)	−0.0002 (14)
O3	0.0244 (14)	0.0199 (15)	0.0229 (15)	−0.0087 (12)	0.0082 (11)	−0.0059 (12)
C6	0.0210 (19)	0.0128 (19)	0.0185 (19)	0.0021 (15)	−0.0010 (15)	−0.0025 (16)
C7	0.030 (2)	0.025 (2)	0.023 (2)	−0.0080 (18)	0.0048 (17)	−0.0107 (18)
C8	0.036 (2)	0.023 (2)	0.020 (2)	−0.0042 (18)	0.0098 (18)	−0.0042 (18)
Br1	0.0302 (2)	0.0259 (2)	0.0255 (2)	0.00937 (17)	−0.00436 (17)	−0.00550 (18)
Co1	0.0140 (2)	0.0128 (3)	0.0133 (3)	−0.00413 (19)	0.00139 (18)	−0.0009 (2)
C11	0.0146 (17)	0.0111 (18)	0.0171 (19)	0.0004 (14)	0.0047 (15)	−0.0014 (15)
O11	0.0190 (13)	0.0231 (15)	0.0241 (15)	−0.0054 (11)	−0.0014 (11)	−0.0065 (12)
C12	0.023 (2)	0.019 (2)	0.0168 (19)	−0.0116 (16)	0.0023 (16)	−0.0030 (16)
O12	0.0360 (17)	0.0303 (17)	0.0251 (16)	−0.0105 (14)	0.0129 (13)	−0.0111 (14)
C13	0.024 (2)	0.027 (2)	0.024 (2)	−0.0126 (18)	−0.0019 (17)	−0.0033 (18)
O13	0.0362 (17)	0.0196 (17)	0.046 (2)	−0.0045 (14)	−0.0146 (15)	0.0100 (15)
Co2	0.0140 (2)	0.0139 (3)	0.0161 (3)	−0.00375 (19)	−0.00161 (19)	−0.0012 (2)
C21	0.0197 (19)	0.023 (2)	0.021 (2)	−0.0035 (17)	−0.0066 (16)	−0.0010 (17)
O21	0.0254 (15)	0.0375 (19)	0.0351 (18)	−0.0164 (14)	−0.0022 (13)	0.0050 (15)
C22	0.0204 (19)	0.020 (2)	0.025 (2)	−0.0062 (16)	−0.0066 (16)	−0.0017 (18)
O22	0.0389 (18)	0.0333 (18)	0.0346 (18)	−0.0058 (14)	−0.0004 (14)	−0.0193 (15)
C23	0.0174 (19)	0.025 (2)	0.025 (2)	−0.0101 (17)	0.0015 (16)	−0.0041 (18)
O23	0.0240 (15)	0.0204 (16)	0.0382 (18)	−0.0019 (13)	−0.0105 (13)	0.0068 (13)

### (1) [µ2-η2,η2-4-Hydroxybut-2-yn-1-yl 2-bromo-2-methylpropanoate-κ4C2,C3:C2,C3]bis[tricarbonylcobalt(II)](Co—Co) Geometric parameters (Å, º)

**Table:**

O1—C1	1.430 (4)	C7—H7A	0.9800
O1—H1	0.8400	C7—H7B	0.9800
C1—C2	1.493 (5)	C7—H7C	0.9800
C1—H1A	0.9900	C8—H8A	0.9800
C1—H1B	0.9900	C8—H8B	0.9800
C2—C3	1.344 (5)	C8—H8C	0.9800
Co1—Co2	2.4723 (7)	Co1—C11	1.805 (4)
C2—Co1	1.967 (3)	Co1—C12	1.819 (4)
C2—Co2	1.972 (3)	Co1—C13	1.833 (4)
C3—C4	1.476 (5)	C11—O11	1.121 (4)
C3—Co1	1.956 (4)	C12—O12	1.141 (5)
C3—Co2	1.960 (3)	C13—O13	1.125 (5)
C4—O2	1.455 (4)	Co2—C21	1.794 (4)
C4—H4A	0.9900	Co2—C22	1.824 (4)
C4—H4B	0.9900	Co2—C23	1.825 (4)
O2—C5	1.331 (4)	C21—O21	1.136 (5)
C5—O3	1.207 (4)	C22—O22	1.135 (5)
C5—C6	1.535 (5)	C23—O23	1.132 (4)
C6—C7	1.516 (5)	Br1—O1^i^	3.307 (3)
C6—C8	1.526 (5)	O2—O21^ii^	2.965 (4)
C6—Br1	1.981 (3)		
			
C1—O1—H1	109.5	C6—C8—H8A	109.5
O1—C1—C2	111.1 (3)	C6—C8—H8B	109.5
O1—C1—H1A	109.4	H8A—C8—H8B	109.5
C2—C1—H1A	109.4	C6—C8—H8C	109.5
O1—C1—H1B	109.4	H8A—C8—H8C	109.5
C2—C1—H1B	109.4	H8B—C8—H8C	109.5
H1A—C1—H1B	108.0	C6—Br1—O1^iii^	68.68 (12)
C3—C2—C1	140.2 (3)	C11—Co1—C12	99.92 (16)
C3—C2—Co1	69.5 (2)	C11—Co1—C13	98.37 (16)
C1—C2—Co1	135.5 (3)	C12—Co1—C13	106.52 (17)
C3—C2—Co2	69.5 (2)	C11—Co1—C3	100.69 (15)
C1—C2—Co2	135.7 (2)	C12—Co1—C3	102.34 (16)
Co1—C2—Co2	77.75 (13)	C13—Co1—C3	141.85 (16)
C2—C3—C4	138.8 (3)	C11—Co1—C2	98.44 (15)
C2—C3—Co1	70.4 (2)	C12—Co1—C2	140.87 (16)
C4—C3—Co1	135.6 (2)	C13—Co1—C2	104.58 (16)
C2—C3—Co2	70.5 (2)	C3—Co1—C2	40.06 (14)
C4—C3—Co2	135.3 (3)	C11—Co1—Co2	148.11 (11)
Co1—C3—Co2	78.29 (13)	C12—Co1—Co2	100.47 (12)
O2—C4—C3	111.1 (3)	C13—Co1—Co2	99.02 (12)
O2—C4—H4A	109.4	C3—Co1—Co2	50.92 (10)
C3—C4—H4A	109.4	C2—Co1—Co2	51.21 (10)
O2—C4—H4B	109.4	O11—C11—Co1	179.3 (3)
C3—C4—H4B	109.4	O12—C12—Co1	176.8 (3)
H4A—C4—H4B	108.0	O13—C13—Co1	179.3 (3)
C5—O2—C4	117.6 (3)	C21—Co2—C22	101.27 (18)
C5—O2—O21^ii^	142.6 (2)	C21—Co2—C23	101.86 (17)
C4—O2—O21^ii^	89.70 (19)	C22—Co2—C23	104.94 (17)
O3—C5—O2	124.9 (3)	C21—Co2—C3	98.31 (15)
O3—C5—C6	123.7 (3)	C22—Co2—C3	103.15 (15)
O2—C5—C6	111.4 (3)	C23—Co2—C3	141.17 (17)
C7—C6—C8	112.2 (3)	C21—Co2—C2	96.59 (16)
C7—C6—C5	110.8 (3)	C22—Co2—C2	141.37 (15)
C8—C6—C5	114.1 (3)	C23—Co2—C2	104.47 (16)
C7—C6—Br1	109.1 (3)	C3—Co2—C2	39.97 (14)
C8—C6—Br1	107.0 (3)	C21—Co2—Co1	145.74 (12)
C5—C6—Br1	103.0 (2)	C22—Co2—Co1	100.40 (12)
C6—C7—H7A	109.5	C23—Co2—Co1	97.76 (12)
C6—C7—H7B	109.5	C3—Co2—Co1	50.79 (10)
H7A—C7—H7B	109.5	C2—Co2—Co1	51.04 (10)
C6—C7—H7C	109.5	O21—C21—Co2	176.0 (3)
H7A—C7—H7C	109.5	O22—C22—Co2	177.1 (3)
H7B—C7—H7C	109.5	O23—C23—Co2	178.5 (3)
			
O1—C1—C2—C3	30.5 (6)	C3—C4—O2—C5	−106.2 (3)
O1—C1—C2—Co1	−85.6 (4)	C3—C4—O2—O21^ii^	100.4 (3)
O1—C1—C2—Co2	147.0 (3)	C4—O2—C5—O3	3.2 (5)
C1—C2—C3—C4	0.4 (8)	O21^ii^—O2—C5—O3	135.5 (3)
Co1—C2—C3—C4	138.1 (5)	C4—O2—C5—C6	−177.1 (3)
Co2—C2—C3—C4	−137.7 (5)	O21^ii^—O2—C5—C6	−44.8 (5)
C1—C2—C3—Co1	−137.7 (5)	O3—C5—C6—C7	22.8 (5)
Co2—C2—C3—Co1	84.12 (10)	O2—C5—C6—C7	−156.8 (3)
C1—C2—C3—Co2	138.1 (5)	O3—C5—C6—C8	150.7 (4)
Co1—C2—C3—Co2	−84.12 (10)	O2—C5—C6—C8	−29.0 (4)
C2—C3—C4—O2	179.8 (4)	O3—C5—C6—Br1	−93.7 (4)
Co1—C3—C4—O2	−64.1 (4)	O2—C5—C6—Br1	86.6 (3)
Co2—C3—C4—O2	64.1 (4)		

Symmetry codes: (i) −*x*+1, −*y*+1, −*z*+2; (ii) *x*+1, *y*, *z*; (iii) −*x*, −*y*+1, −*z*+2.

### (1) [µ2-η2,η2-4-Hydroxybut-2-yn-1-yl 2-bromo-2-methylpropanoate-κ4C2,C3:C2,C3]bis[tricarbonylcobalt(II)](Co—Co) Hydrogen-bond geometry (Å, º)

**Table:**

*D*—H···*A*	*D*—H	H···*A*	*D*···*A*	*D*—H···*A*
O1—H1···O3^iii^	0.84	2.16	2.946 (4)	156
C4—H4*B*···O3^iii^	0.99	2.60	3.360 (4)	134
C7—H7*A*···O1^iii^	0.98	2.71	3.637 (5)	157
C1—H1*A*···O1^iv^	0.99	2.55	3.307 (5)	133
C8—H8*A*···O12^v^	0.98	2.71	3.485 (5)	136

Symmetry codes: (iii) −*x*, −*y*+1, −*z*+2; (iv) −*x*, −*y*+2, −*z*+2; (v) −*x*+1, −*y*+1, −*z*+1.

### (2) [µ2-η2,η2-But-2-yne-1,4-diyl bis(2-bromo-2-methylpropanoate)-κ4C2,C3:C2,C3]bis[tricarbonylcobalt(II)](Co—Co) Crystal data

**Table:**

[Co_2_(C_12_H_16_Br_2_O_4_)(CO)_6_]	*Z* = 2
*M**_r_* = 669.99	*F*(000) = 656
Triclinic, *P*1	*D*_x_ = 1.851 Mg m^−^^3^
*a* = 9.392 (5) Å	Mo *K*α radiation, λ = 0.71069 Å
*b* = 10.710 (5) Å	Cell parameters from 5837 reflections
*c* = 13.269 (5) Å	θ = 2.3–30.9°
α = 71.314 (5)°	µ = 4.75 mm^−^^1^
β = 71.973 (5)°	*T* = 91 K
γ = 84.630 (5)°	Rod, dark red
*V* = 1202.3 (10) Å^3^	0.25 × 0.11 × 0.06 mm

### (2) [µ2-η2,η2-But-2-yne-1,4-diyl bis(2-bromo-2-methylpropanoate)-κ4C2,C3:C2,C3]bis[tricarbonylcobalt(II)](Co—Co) Data collection

**Table:**

Bruker APEXII CCD area-detector diffractometer	6040 reflections with *I* > 2σ(*I*)
Radiation source: fine-focus sealed tube	*R*_int_ = 0.037
ω scans	θ_max_ = 32.3°, θ_min_ = 1.7°
Absorption correction: multi-scan (SADABS; Bruker, 2011)	*h* = −14→12
*T*_min_ = 0.611, *T*_max_ = 1.000	*k* = −16→16
21546 measured reflections	*l* = −19→18
8127 independent reflections	

### (2) [µ2-η2,η2-But-2-yne-1,4-diyl bis(2-bromo-2-methylpropanoate)-κ4C2,C3:C2,C3]bis[tricarbonylcobalt(II)](Co—Co) Refinement

**Table:**

Refinement on *F*^2^	0 restraints
Least-squares matrix: full	Hydrogen site location: inferred from neighbouring sites
*R*[*F*^2^ > 2σ(*F*^2^)] = 0.031	H-atom parameters constrained
*wR*(*F*^2^) = 0.069	*w* = 1/[σ^2^(*F*_o_^2^) + (0.0311*P*)^2^] where *P* = (*F*_o_^2^ + 2*F*_c_^2^)/3
*S* = 0.95	(Δ/σ)_max_ = 0.008
8127 reflections	Δρ_max_ = 1.43 e Å^−^^3^
293 parameters	Δρ_min_ = −1.06 e Å^−^^3^

### (2) [µ2-η2,η2-But-2-yne-1,4-diyl bis(2-bromo-2-methylpropanoate)-κ4C2,C3:C2,C3]bis[tricarbonylcobalt(II)](Co—Co) Special details

**Table:**

Geometry. All e.s.d.'s (except the e.s.d. in the dihedral angle between two l.s. planes) are estimated using the full covariance matrix. The cell e.s.d.'s are taken into account individually in the estimation of e.s.d.'s in distances, angles and torsion angles; correlations between e.s.d.'s in cell parameters are only used when they are defined by crystal symmetry. An approximate (isotropic) treatment of cell e.s.d.'s is used for estimating e.s.d.'s involving l.s. planes.

### (2) [µ2-η2,η2-But-2-yne-1,4-diyl bis(2-bromo-2-methylpropanoate)-κ4C2,C3:C2,C3]bis[tricarbonylcobalt(II)](Co—Co) Fractional atomic coordinates and isotropic or equivalent isotropic displacement parameters (Å^2^)

**Table:**

	*x*	*y*	*z*	*U*_iso_*/*U*_eq_	
Br1	0.75011 (2)	0.49577 (2)	0.81786 (2)	0.02064 (5)	
C7	0.6143 (2)	0.74032 (18)	0.72699 (17)	0.0192 (4)	
H7A	0.5332	0.7891	0.6996	0.029*	
H7B	0.7079	0.7554	0.6657	0.029*	
H7C	0.6260	0.7708	0.7862	0.029*	
C6	0.4399 (2)	0.56200 (19)	0.87435 (16)	0.0167 (4)	
H6A	0.3511	0.6010	0.8526	0.025*	
H6B	0.4533	0.5981	0.9300	0.025*	
H6C	0.4270	0.4662	0.9061	0.025*	
C5	0.5769 (2)	0.59405 (18)	0.77269 (16)	0.0140 (4)	
C4	0.5703 (2)	0.54055 (17)	0.68077 (16)	0.0134 (4)	
O2	0.63231 (16)	0.58983 (13)	0.58281 (11)	0.0182 (3)	
O1	0.48633 (15)	0.43066 (12)	0.72283 (11)	0.0140 (3)	
C3	0.4766 (2)	0.36816 (18)	0.64290 (15)	0.0143 (4)	
H3A	0.5769	0.3390	0.6063	0.017*	
H3B	0.4373	0.4311	0.5848	0.017*	
C2	0.3747 (2)	0.25397 (17)	0.70430 (15)	0.0126 (4)	
C1	0.3246 (2)	0.15033 (18)	0.68869 (15)	0.0123 (4)	
C8	0.3382 (2)	0.09321 (18)	0.59884 (16)	0.0147 (4)	
H8A	0.3121	0.1598	0.5358	0.018*	
H8B	0.4424	0.0644	0.5715	0.018*	
O3	0.23609 (15)	−0.01940 (12)	0.64364 (11)	0.0149 (3)	
C9	0.2214 (2)	−0.07240 (18)	0.56873 (16)	0.0142 (4)	
O4	0.28657 (18)	−0.03238 (14)	0.47087 (12)	0.0232 (3)	
C10	0.1107 (2)	−0.18786 (18)	0.62156 (17)	0.0151 (4)	
C11	0.0394 (3)	−0.2029 (2)	0.53826 (19)	0.0235 (5)	
H11A	−0.0159	−0.2866	0.5690	0.035*	
H11B	0.1175	−0.2021	0.4690	0.035*	
H11C	−0.0297	−0.1298	0.5228	0.035*	
C12	−0.0002 (3)	−0.1871 (2)	0.7318 (2)	0.0316 (6)	
H12A	−0.0649	−0.1099	0.7201	0.047*	
H12B	0.0541	−0.1835	0.7832	0.047*	
H12C	−0.0615	−0.2675	0.7636	0.047*	
Br2	0.24396 (3)	−0.34166 (2)	0.65043 (2)	0.03253 (7)	
Co1	0.37507 (3)	0.09639 (2)	0.82922 (2)	0.01282 (6)	
C13	0.2937 (2)	−0.0691 (2)	0.89243 (17)	0.0205 (4)	
O13	0.2402 (2)	−0.17088 (14)	0.92908 (14)	0.0318 (4)	
C14	0.3706 (2)	0.15581 (19)	0.94411 (17)	0.0204 (4)	
O14	0.3689 (2)	0.19624 (15)	1.01387 (13)	0.0319 (4)	
C15	0.5735 (3)	0.06630 (19)	0.78595 (17)	0.0192 (4)	
O15	0.69906 (18)	0.05518 (17)	0.75817 (14)	0.0304 (4)	
Co2	0.16116 (3)	0.22826 (2)	0.78080 (2)	0.01303 (6)	
C16	0.0196 (2)	0.0985 (2)	0.84785 (17)	0.0179 (4)	
O16	−0.06494 (18)	0.01443 (15)	0.88945 (13)	0.0269 (4)	
C17	0.1197 (2)	0.3291 (2)	0.87509 (19)	0.0214 (4)	
O17	0.0949 (2)	0.39023 (17)	0.93381 (15)	0.0367 (4)	
C18	0.0866 (2)	0.3323 (2)	0.67223 (18)	0.0203 (4)	
O18	0.0423 (2)	0.39556 (16)	0.60153 (14)	0.0316 (4)	

### (2) [µ2-η2,η2-But-2-yne-1,4-diyl bis(2-bromo-2-methylpropanoate)-κ4C2,C3:C2,C3]bis[tricarbonylcobalt(II)](Co—Co) Atomic displacement parameters (Å^2^)

**Table:**

	*U*^11^	*U*^22^	*U*^33^	*U*^12^	*U*^13^	*U*^23^
Br1	0.01709 (11)	0.02490 (11)	0.02151 (11)	−0.00005 (8)	−0.00881 (8)	−0.00622 (8)
C7	0.0267 (12)	0.0152 (9)	0.0180 (10)	−0.0055 (8)	−0.0077 (8)	−0.0054 (8)
C6	0.0178 (10)	0.0187 (9)	0.0128 (9)	−0.0030 (8)	−0.0027 (7)	−0.0047 (7)
C5	0.0169 (10)	0.0136 (8)	0.0136 (9)	−0.0020 (7)	−0.0076 (7)	−0.0034 (7)
C4	0.0150 (10)	0.0116 (8)	0.0143 (9)	−0.0023 (7)	−0.0058 (7)	−0.0031 (7)
O2	0.0226 (8)	0.0179 (7)	0.0127 (7)	−0.0087 (6)	−0.0023 (6)	−0.0031 (5)
O1	0.0181 (7)	0.0130 (6)	0.0118 (6)	−0.0078 (5)	−0.0031 (5)	−0.0041 (5)
C3	0.0191 (10)	0.0138 (8)	0.0114 (9)	−0.0060 (7)	−0.0036 (7)	−0.0051 (7)
C2	0.0122 (9)	0.0124 (8)	0.0128 (9)	−0.0011 (7)	−0.0033 (7)	−0.0035 (7)
C1	0.0105 (9)	0.0135 (8)	0.0124 (9)	−0.0008 (7)	−0.0026 (7)	−0.0038 (7)
C8	0.0146 (10)	0.0147 (8)	0.0136 (9)	−0.0066 (7)	−0.0006 (7)	−0.0043 (7)
O3	0.0168 (7)	0.0145 (6)	0.0134 (7)	−0.0068 (5)	−0.0011 (5)	−0.0056 (5)
C9	0.0129 (9)	0.0141 (8)	0.0190 (10)	0.0017 (7)	−0.0070 (7)	−0.0077 (7)
O4	0.0279 (9)	0.0270 (8)	0.0163 (7)	−0.0110 (7)	−0.0035 (6)	−0.0086 (6)
C10	0.0140 (10)	0.0123 (8)	0.0207 (10)	0.0002 (7)	−0.0067 (8)	−0.0059 (7)
C11	0.0216 (12)	0.0227 (10)	0.0312 (12)	−0.0052 (9)	−0.0156 (9)	−0.0059 (9)
C12	0.0267 (13)	0.0314 (12)	0.0347 (13)	−0.0165 (10)	0.0075 (10)	−0.0190 (11)
Br2	0.03671 (15)	0.01571 (10)	0.05628 (17)	0.00796 (9)	−0.03120 (13)	−0.01135 (10)
Co1	0.01475 (14)	0.01102 (12)	0.01208 (12)	−0.00128 (10)	−0.00391 (10)	−0.00241 (9)
C13	0.0217 (11)	0.0184 (10)	0.0198 (10)	0.0038 (8)	−0.0049 (8)	−0.0061 (8)
O13	0.0383 (10)	0.0140 (7)	0.0350 (9)	−0.0047 (7)	−0.0020 (8)	−0.0037 (7)
C14	0.0262 (12)	0.0153 (9)	0.0186 (10)	−0.0013 (8)	−0.0096 (9)	−0.0005 (8)
O14	0.0538 (12)	0.0281 (8)	0.0198 (8)	0.0015 (8)	−0.0169 (8)	−0.0103 (7)
C15	0.0238 (12)	0.0183 (9)	0.0152 (9)	0.0005 (8)	−0.0089 (8)	−0.0019 (8)
O15	0.0179 (9)	0.0433 (10)	0.0290 (9)	0.0048 (7)	−0.0082 (7)	−0.0100 (7)
Co2	0.01267 (13)	0.01312 (12)	0.01367 (13)	−0.00069 (10)	−0.00305 (10)	−0.00523 (10)
C16	0.0151 (10)	0.0245 (10)	0.0152 (9)	0.0001 (8)	−0.0015 (8)	−0.0102 (8)
O16	0.0219 (9)	0.0329 (9)	0.0241 (8)	−0.0118 (7)	0.0022 (7)	−0.0117 (7)
C17	0.0194 (11)	0.0220 (10)	0.0252 (11)	0.0015 (8)	−0.0068 (9)	−0.0108 (9)
O17	0.0380 (11)	0.0400 (10)	0.0421 (11)	0.0063 (8)	−0.0098 (8)	−0.0299 (9)
C18	0.0180 (11)	0.0202 (10)	0.0251 (11)	0.0012 (8)	−0.0053 (9)	−0.0114 (8)
O18	0.0359 (10)	0.0342 (9)	0.0285 (9)	0.0105 (7)	−0.0173 (8)	−0.0102 (7)

### (2) [µ2-η2,η2-But-2-yne-1,4-diyl bis(2-bromo-2-methylpropanoate)-κ4C2,C3:C2,C3]bis[tricarbonylcobalt(II)](Co—Co) Geometric parameters (Å, º)

**Table:**

Br1—C5	1.998 (2)	O3—C9	1.338 (2)
C7—C5	1.519 (3)	C9—O4	1.202 (2)
C7—H7A	0.9800	C9—C10	1.528 (3)
C7—H7B	0.9800	C10—C11	1.513 (3)
C7—H7C	0.9800	C10—C12	1.513 (3)
C6—C5	1.518 (3)	C10—Br2	1.983 (2)
C6—H6A	0.9800	C11—H11A	0.9800
C6—H6B	0.9800	C11—H11B	0.9800
C6—H6C	0.9800	C11—H11C	0.9800
C5—C4	1.524 (3)	C12—H12A	0.9800
C4—O2	1.207 (2)	C12—H12B	0.9800
C4—O1	1.341 (2)	C12—H12C	0.9800
O1—C3	1.452 (2)	Co1—C15	1.803 (2)
C3—C2	1.474 (3)	Co1—C14	1.819 (2)
C3—H3A	0.9900	Co1—C13	1.826 (2)
C3—H3B	0.9900	C13—O13	1.135 (2)
C1—C2	1.343 (3)	C14—O14	1.136 (3)
Co1—Co2	2.4759 (10)	C15—O15	1.128 (3)
C1—Co1	1.960 (2)	Co2—C18	1.805 (2)
C1—Co2	1.949 (2)	Co2—C16	1.820 (2)
C2—Co1	1.9508 (19)	Co2—C17	1.835 (2)
C2—Co2	1.948 (2)	C16—O16	1.136 (2)
C1—C8	1.473 (3)	C17—O17	1.130 (3)
C8—O3	1.460 (2)	C18—O18	1.137 (3)
C8—H8A	0.9900	O1—Br2^i^	3.2960 (18)
C8—H8B	0.9900		
			
C5—C7—H7A	109.5	C11—C10—C9	110.93 (16)
C5—C7—H7B	109.5	C12—C10—C9	114.10 (17)
H7A—C7—H7B	109.5	C11—C10—Br2	106.58 (14)
C5—C7—H7C	109.5	C12—C10—Br2	107.91 (15)
H7A—C7—H7C	109.5	C9—C10—Br2	102.33 (13)
H7B—C7—H7C	109.5	C10—C11—H11A	109.5
C5—C6—H6A	109.5	C10—C11—H11B	109.5
C5—C6—H6B	109.5	H11A—C11—H11B	109.5
H6A—C6—H6B	109.5	C10—C11—H11C	109.5
C5—C6—H6C	109.5	H11A—C11—H11C	109.5
H6A—C6—H6C	109.5	H11B—C11—H11C	109.5
H6B—C6—H6C	109.5	C10—C12—H12A	109.5
C6—C5—C7	112.32 (17)	C10—C12—H12B	109.5
C6—C5—C4	114.29 (16)	H12A—C12—H12B	109.5
C7—C5—C4	111.00 (15)	C10—C12—H12C	109.5
C6—C5—Br1	107.83 (13)	H12A—C12—H12C	109.5
C7—C5—Br1	108.67 (14)	H12B—C12—H12C	109.5
C4—C5—Br1	102.04 (13)	C15—Co1—C14	97.61 (10)
O2—C4—O1	124.07 (18)	C15—Co1—C13	103.39 (10)
O2—C4—C5	124.81 (17)	C14—Co1—C13	106.12 (9)
O1—C4—C5	111.12 (15)	C15—Co1—C2	96.50 (8)
C4—O1—C3	115.91 (14)	C14—Co1—C2	105.58 (9)
C4—O1—Br2^i^	78.24 (11)	C13—Co1—C2	139.62 (9)
C3—O1—Br2^i^	91.57 (10)	C15—Co1—C1	102.92 (9)
O1—C3—C2	107.52 (15)	C14—Co1—C1	141.32 (9)
O1—C3—H3A	110.2	C13—Co1—C1	100.56 (9)
C2—C3—H3A	110.2	C2—Co1—C1	40.16 (8)
O1—C3—H3B	110.2	C15—Co1—Co2	146.69 (6)
C2—C3—H3B	110.2	C14—Co1—Co2	96.39 (7)
H3A—C3—H3B	108.5	C13—Co1—Co2	101.48 (8)
C1—C2—C3	140.49 (18)	C2—Co1—Co2	50.52 (6)
C1—C2—Co2	69.89 (11)	C1—Co1—Co2	50.50 (6)
C3—C2—Co2	135.87 (14)	O13—C13—Co1	177.5 (2)
C1—C2—Co1	70.29 (11)	O14—C14—Co1	178.14 (18)
C3—C2—Co1	133.59 (14)	O15—C15—Co1	175.85 (19)
Co2—C2—Co1	78.85 (7)	C18—Co2—C16	100.32 (10)
C2—C1—C8	139.74 (17)	C18—Co2—C17	100.21 (10)
C2—C1—Co2	69.80 (12)	C16—Co2—C17	104.28 (10)
C8—C1—Co2	134.45 (14)	C18—Co2—C2	99.95 (9)
C2—C1—Co1	69.55 (11)	C16—Co2—C2	140.82 (9)
C8—C1—Co1	136.33 (14)	C17—Co2—C2	104.64 (9)
Co2—C1—Co1	78.60 (8)	C18—Co2—C1	98.17 (9)
O3—C8—C1	108.09 (15)	C16—Co2—C1	103.58 (9)
O3—C8—H8A	110.1	C17—Co2—C1	143.05 (9)
C1—C8—H8A	110.1	C2—Co2—C1	40.31 (8)
O3—C8—H8B	110.1	C18—Co2—Co1	147.24 (7)
C1—C8—H8B	110.1	C16—Co2—Co1	97.95 (8)
H8A—C8—H8B	108.4	C17—Co2—Co1	101.33 (7)
C9—O3—C8	115.40 (14)	C2—Co2—Co1	50.63 (6)
O4—C9—O3	123.67 (18)	C1—Co2—Co1	50.90 (6)
O4—C9—C10	124.03 (18)	O16—C16—Co2	177.62 (19)
O3—C9—C10	112.30 (16)	O17—C17—Co2	179.3 (2)
C11—C10—C12	113.98 (19)	O18—C18—Co2	177.7 (2)
			
C6—C5—C4—O2	151.45 (19)	Co1—C2—C1—C8	−139.0 (3)
C7—C5—C4—O2	23.2 (3)	C3—C2—C1—Co2	−139.2 (3)
Br1—C5—C4—O2	−92.5 (2)	Co1—C2—C1—Co2	84.98 (6)
C6—C5—C4—O1	−28.7 (2)	C3—C2—C1—Co1	135.8 (3)
C7—C5—C4—O1	−156.98 (16)	Co2—C2—C1—Co1	−84.98 (6)
Br1—C5—C4—O1	87.39 (16)	C2—C1—C8—O3	−174.7 (2)
O2—C4—O1—C3	2.0 (3)	Co2—C1—C8—O3	−60.5 (2)
C5—C4—O1—C3	−177.82 (15)	Co1—C1—C8—O3	68.3 (2)
O2—C4—O1—Br2^i^	−83.95 (19)	C1—C8—O3—C9	172.04 (16)
C5—C4—O1—Br2^i^	96.19 (14)	C8—O3—C9—O4	0.2 (3)
C4—O1—C3—C2	−177.40 (15)	C8—O3—C9—C10	−178.94 (15)
Br2^i^—O1—C3—C2	−99.72 (14)	O4—C9—C10—C11	−26.6 (3)
O1—C3—C2—C1	−173.2 (2)	O3—C9—C10—C11	152.47 (17)
O1—C3—C2—Co2	68.6 (2)	O4—C9—C10—C12	−157.0 (2)
O1—C3—C2—Co1	−58.2 (2)	O3—C9—C10—C12	22.1 (2)
C3—C2—C1—C8	−3.2 (5)	O4—C9—C10—Br2	86.7 (2)
Co2—C2—C1—C8	136.1 (3)	O3—C9—C10—Br2	−94.18 (16)

Symmetry code: (i) *x*, *y*+1, *z*.

### (2) [µ2-η2,η2-But-2-yne-1,4-diyl bis(2-bromo-2-methylpropanoate)-κ4C2,C3:C2,C3]bis[tricarbonylcobalt(II)](Co—Co) Hydrogen-bond geometry (Å, º)

**Table:**

*D*—H···*A*	*D*—H	H···*A*	*D*···*A*	*D*—H···*A*
C12—H12*C*···Br1^ii^	0.98	2.99	3.961 (3)	170
C6—H6*A*···Br2^i^	0.98	3.01	3.788 (2)	137
C8—H8*B*···O4^iii^	0.99	2.45	3.411 (3)	165
C3—H3*B*···O2^iv^	0.99	2.58	3.341 (3)	133
C8—H8*A*···O2^iv^	0.99	2.64	3.454 (3)	139

Symmetry codes: (i) *x*, *y*+1, *z*; (ii) *x*−1, *y*−1, *z*; (iii) −*x*+1, −*y*, −*z*+1; (iv) −*x*+1, −*y*+1, −*z*+1.
